# Is glycaemic control essential for cataract surgery among patients with diabetes mellitus?

**DOI:** 10.6026/973206300211011

**Published:** 2025-05-31

**Authors:** Ramachandra Himateja C., Sangeetha T., Inchara N.

**Affiliations:** 1 Department of Ophthalmology, Sri Devaraj Urs Medical college,Tamaka, Kolar, Karnataka, India

**Keywords:** Cataract surgery, diabetes mellitus, HbA1c, glycaemic control, post-operative complications, diabetic retinopathy, macular edema

## Abstract

Diabetic patients are at increased risk of cataracts, with higher risks of intraoperative and postoperative complications. Therefore, it is of interest to explore the challenges in performing cataract surgery on diabetic patients. Hence, a prospective study of 300 Type II diabetes patients undergoing cataract surgery found that 69.3% achieved post-operative visual acuity of 6/18 or better. Common complications included iritis (9.7%) and striate keratopathy (7.7%). The severity of diabetic retinopathy, diabetic macular edema, and poor preoperative visual acuity were linked to worse postoperative outcomes. Intravitreal steroids may improve outcomes in patients with diabetic macular edema.

## Background:

Cataract surgery is the most commonly performed intraocular procedure in ophthalmology, aimed at restoring vision. Diabetes mellitus (DM) is one of the most widespread and debilitating chronic conditions, affecting millions of individuals globally. Diabetic patients are at an increased risk of developing cataracts, particularly cortical and posterior sub-capsular opacities, which often result in them seeking surgery at a younger age. The likelihood of cataract formation escalates with the duration of diabetes, the severity of hyperglycaemia, and the patient's age [1]. Cataract surgery in diabetic patients is associated with a higher risk of both intraoperative and postoperative complications compared to non-diabetic individuals [2, 3]. Individuals with inadequately controlled diabetes are frequently deemed unfit for surgical procedures due to the elevated risk of postoperative complications, including impaired wound healing and an increased susceptibility to infections [4]. Some research has suggested that cataract surgery in these patients may accelerate the progression of diabetic retinopathy (DR), trigger vitreous haemorrhage, induce iris neovascularization and potentially result in diminished or total loss of vision [5]. According to the Early Treatment Diabetic Retinopathy Study (ETDRS), the presence of clinically significant macular edema (CSME) and the severity of DR at the time of cataract surgery are key predictors of poor postoperative visual acuity. Advanced DR is linked to macular ischemia, persistent macular edema and increased risk of complications such as vitreous haemorrhage and tractional retinal detachment, especially in untreated proliferative DR [6]. However, there are no definitive guidelines regarding the HbA1c range at which cataract surgery is considered safe, nor is there clarity on how to prevent postoperative complications or the advancement of retinopathy and maculopathy. Therefore, it is of interest to explore the challenges of performing cataract surgery on diabetic patients.

## Materials and Methods:

This prospective hospital-based study was conducted on 300 cataract patients with diabetes mellitus of more 10 years duration in the department of Ophthalmology at a tertiary care centre from July 2023 to June 2024 after obtaining approval from the Central Ethics Committee and written informed consent from all patients. Cases with incomplete follow-ups and with history of intraocular surgeries or advanced ocular comorbidities significantly affecting visual outcomes were excluded. Pre-operative data includes demographic data, slit lamp examination to evaluate the anterior segment and indirect ophthalmoscopy to assess the posterior segment for diabetic retinopathy graded as per the ETDRS classification, [7] medical co-morbidities (diabetes, hypertension and coronary heart disease), and use of any medications with dose and duration followed by haematological investigations such as fasting sugar, postprandial blood sugar and glycated haemoglobin levels. Physician opinion was sought for patients with deranged sugars prior to surgery irrespective of the glycated haemoglobin levels. The standard preoperative regime included topical flurbiprofen (0.03% w/v) or nepafenac (0.1% w/v), two hours before surgery followed by pupillary dilatation using topical tropicamide (0.8%w/v) and phenylephrine (5%w/v). Routine small incision cataract surgery was performed under peribulbar anaesthesia. Preoperative and postoperative visual acuity, intraoperative and postoperative complications were noted and correlated with the glycaemic status. Data is analysed using SPSS version 22 software with p-value of < 0.05 was considered statistically significant.

## Results and Discussion:

Table 1 shows the characteristics of 300 participants 154 (51.3%) male and 146 (48.7%) females were included in this study. The mean age of the participants is 62.16 ± 10.29 [ranged 30- 81] years. The duration of diabetes was 14.61 ± 4.5 years. The mean HbA1c level was 8.63+2.12% (range 5.0-15.7).The number of patients with history of DM < 10 years and > 10 years duration were 242 (80.7%) and 58 (19.3%) respectively. The mean Fasting Blood Glucose and Post Prandial Blood Glucose were 133.3 ± 64.7 and 195.2 ± 88.4 in the 1st group of 242 patients with a mean HbA1c of 8.2+2.1. The mean Fasting Blood Glucose and Post Prandial Blood Glucose were 127.5+54.4 and 206.2+91.4 in the 2nd group of 58 patients with a mean HbA1c of 8.5 ± 2.3. Patients with glycated haemoglobin of <6.5% were 39 (13%) and > 6.5 % were 261 (87%) with a mean HbA1c of 8.63 ± 2.12% (range 5.0-15.7). Table 2 displays the stratification of visual acuity. Good visual outcome of 6/18 or better was noted in 208 (69.3%), while 42 (14%) had 6/36 or better and 50 (16.6%) had 6/60. The mean Preoperative and postoperative BCVA was 0.91+ 0.75 & 0.43+ 0.42 (p < 0.01). Table 3 reveals the statistically significant correlation between the postoperative visual outcomes with the glycated haemoglobin levels. Good vision was observed in 173 (66.3%) patients' moderate vision in 42 (16.1%) patients even with HbA1c of > 6.5%.The common immediate post-operative complications encountered were Iritis in 29 (9.7%) patients and Striate Keratopathy in 23 (7.7%) patients. Posterior Capsular Rent occurred in 14 (4.7%) patients who were managed by performing anterior vitrectomy followed by iris claw IOL implantation in 7 cases and sulcus implanted IOL in 9 cases. Transient raised IOP was encountered in 9 (3%) patients who were managed medically with IOP lowering medications. Iris Prolapse was encountered in 3 (1%) patients who were managed intra-operatively and 1 (0.3%) patient ended up with endophthalmitis for which he was referred to Vitreo- Retinal consultant for further management. Table 4 highlights the different stages of DR, ocular risk factors and treatment patterns across DR stages. While 72.7% had no DR-suggesting early disease or good glycaemic control-21.3% showed varying degrees of NPDR and 6% had PDR, indicating progression in a minority. (Figure 1 & 2 see PDF) Hard cataract was the most common association (45.34%), followed by glaucoma (14%) and small pupil (12.3%), all of which can affect visual outcomes and surgical planning. Less frequent but notable findings included pseudo exfoliation (9.4%) and ARMD (2%). Treatment patterns reflected current standards, with pan retinal photocoagulation (10.3%) and anti-VEGF injections (8.0%) commonly used, while triamcinolone (2.3%) was less favoured due to potential side effects.

The present study reports a near-equal gender distribution (51.3% male, 48.7% female) among diabetic patients undergoing cataract surgery, with a majority (60.7%) aged over 60 years. This aligns with findings from the Sankara Nethralaya Diabetic Retinopathy Epidemiology and Molecular Genetics Study, which identified increasing age as a significant risk factor for cataract development in diabetic individuals [8]. Similarly, a study by Naik and Joshi observed a higher prevalence of cataracts in older diabetic patients [9]. In this cohort, 80.7% had diabetes for less than 10 years, yet 87% exhibited HbA1c levels above 6.5%, indicating suboptimal glycaemic control. This is consistent with the findings of the SankaraNethralaya study, which reported that poor glycaemic control, as indicated by elevated HbA1c levels, is a significant risk factor for cataract formation in diabetic patients [10]. Additionally, the study by Naik and Joshi emphasized the importance of glycaemic control in the management of diabetic patients undergoing cataract surgery. The high prevalence of poor glycaemic control among patients with a relatively short duration of diabetes underscores the need for early and aggressive management of blood glucose levels to prevent ocular complications. No studies define specific blood glucose thresholds for delaying elective surgery, but perioperative hyperglycaemia (>140-180 mg/dL or >7.8-10.0 mmol/L) is linked to increased postoperative complications in non-cataract, non-cardiac surgeries [11]. Regular ophthalmic evaluations are crucial for early detection and management of cataracts in diabetic patients. A significant improvement in visual acuity was noted post-cataract surgery among diabetic patients. Preoperatively, 93% of patients had visual acuity worse than 6/60, which reduced to 16.6% postoperatively. Additionally, 69.3% achieved visual acuity of 6/18 or better after surgery, with a statistically significant mean improvement from 0.9184 to 0.4269 (p < 0.01). These findings align with those of Naik and Joshi, who reported that 31.7% of diabetic patients achieved a BCVA of 6/9 or better six weeks post-phacoemulsification surgery [12].

Similarly, a study by Sowmya and Vallabha found that 62.1% of diabetic patients attained postoperative visual acuity of 6/12 or better following small incision cataract surgery [13]. Further supporting these outcomes, a study by Shaikh *et al.* observed that diabetic patients without diabetic retinopathy achieved a mean BCVA improvement from 0.81 ± 0.18 preoperatively to 0.18 ± 0.14 six months postoperatively [14]. However, the presence and severity of DR can influence postoperative visual outcomes. Eyes with no DR or with mild to moderate NPDR demonstrated significantly greater improvements in VA one year postoperatively, compared to eyes with severe NPDR or PDR, after adjusting for baseline VA (P < 0.001) [15]. Interestingly, the ACCORD study found that while diabetic retinopathy status was associated with visual outcomes post-cataract surgery, factors such as HbA1c levels were not significantly correlated with achieving a good visual outcome [16]. This suggests that the visual prognosis after cataract surgery is generally favourable for people with diabetes. Previous studies have reported that between 62% and 89% of individuals with diabetes experience a good visual outcome. For diabetic patients, the presence or absence of DR and its severity is particularly important in determining visual outcomes. This study also demonstrates a statistically significant association between glycaemic control and postoperative visual acuity in diabetic patients undergoing cataract surgery (p = 0.032). Patients with HbA1c levels below 6.5% achieved better visual outcomes, with 41% attaining 6/6-6/9 vision, compared to 30.7% in those with higher HbA1c levels. Conversely, a higher percentage of patients with poor glycaemic control had VA worse than 6/60 (17.6% vs. 10.3%). Similarly, a multicentre study by Lundström *et al.* found that diabetic patients had a mean postoperative VA of 0.23 logMAR, slightly worse than non-diabetic patients, and a higher rate of intraoperative complications [17]. However, other studies suggest that HbA1c levels may not be a significant predictor of postoperative visual outcomes. The ACCORD Eye Study concluded that while DR status was associated with visual outcomes, HbA1c levels were not significantly correlated [16]. Similarly, a large retrospective study by Chen *et al.* involving over 65,000 patients found no significant association between preoperative HbA1c levels and postoperative visual acuity [18]. However, a Multivariate analysis revealed that HbA1c is an independent risk for post-operative CME with a relative risk of 2.01 when HBa1c is above 7 c (95% CI, 1.10-3.67) [19]. Present study reported immediate postoperative complications such as Iritis (9.7%), Striate Keratopathy (7.7%), Posterior Capsular Rent (PCR, 4.7%), Transient Raised IOP (3%), Iris Prolapse (1%) and Endophthalmitis (0.3%). The most common complication reported by Alsarhani *et al.* was corneal edema (181, 62.4%), followed by astigmatism (84, 29%), diabetic retinopathy (26, 9%), and macular edema (9, 3.1%) [20]. The authors concluded that while glycaemic control did not significantly alter the incidence of intraoperative complications, poorly controlled diabetes was linked to delayed visual recovery and prolonged inflammation postoperatively. Diabetic patients undergoing cataract surgery face a 31% higher risk of endophthalmitis compared to non-diabetic individuals. Additionally, posterior capsular opacification which can be effectively manage with laser treatment. Moreover, cataract surgery may lead to worsening or new onset of diabetic retinopathy [21]. It is noted that patients with pre-existing moderate to severe NPDR and DME and poor glycaemic control are at a higher risk for developing or exacerbating their DME with possible progression of their DR [22]. Pre-existing conditions like macular ischemia, DME and PDR can limit visual outcomes after cataract surgery, which may also worsen DME or diabetic retinopathy. The Joint British Diabetes Societies [23] and the American Diabetes Association [24] recommend maintaining perioperative blood glucose between 140-180 mg/dL (7.8-10.0 mmol/L). Intensive insulin therapy (IIT), targeting near-normal levels (70-110 mg/dL or 3.9-6.1 mmol/L), carries a higher risk of hypoglycaemia, with consequences outweighing its benefits. Timing of surgery must balance visual improvement with retinal visualization and timely laser treatment. While glycaemic control doesn't affect complication rates, it influences recovery duration and severity, highlighting the need for optimized perioperative glycaemic levels.

## Limitations:

The use of a single hospital database may limit the ability to adjust for unknown confounding factors, and this study could not specifically investigate the effects of DME or anti-VEGF injections on postoperative.

## Conclusion:

Poor pre-operative vision, advanced diabetic retinopathy and diabetic macular edema predict limited visual improvement after cataract surgery. While glycaemic control is important, HbA1c levels do not strongly correlate with visual outcomes. In low-resource settings, delaying surgery due to high blood sugar may result in lost follow-up, worsened vision and complications like lens-induced glaucoma. Early surgery with intraoperative intra-vitreal steroids can improve outcomes in DME patients.

## Acronyms/ Abbreviations/ Initialisms:

DM - Diabetes Mellitus

DR - Diabetic Retinopathy

DME - Diabetic Macular Edema

ETDRS - Early Treatment Diabetic Retinopathy Study

HbA1c - Glycated Hemoglobin

NPDR - Non Proliferative Diabetic Retinopathy

PDR - Proliferative Diabetic Retinopathy

PXF - Pseudoexfoliation

ARMD - Age Related Macular Degeneration

VEGF - Vascular Endothelial Growth Factor,

VA - Visual Acuity

BCVA - Best Corrected Visual Acuity

ACCORD - Action to Control Cardiovascular Risk in Diabetes

PCR - Posterior Capsular Rent

IOP - Intra Ocular Pressure

## Figures and Tables

**Table 1:** Characteristics of study participants

**Characteristics**		**Frequency**	**Percent**
Gender	Male	154	51.3
	Female	146	48.7
Age (years)	40	11	3.7
	41-50	19	6.3
	51-60	88	29.3
	>60	182	60.7
Duration of DM (years)	< 10	242	80.7
	> 10	58	19.3
Glycated hemoglobin	< 6.5%	39	13
	> 6.5 %	261	87

**Table 2:** Comparison of preoperative and postoperative visual acuity

**Visual Acuity**	**Pre-op**		**Post-op**	
	N	%	N	%
6/6 to 6/9	0	0	101	33.6
6/12 to 6/18	7	2.3	107	35.7
6/24 to 6/36	14	4.7	42	14
>6/60	279	93	50	16.6
Mean	0.9184		0.4269	
SD	0.74213		0.42017	
P Value	< 0.01			

**Table 3:** Correlation of postoperative visual acuity with HbA1c levels

**Post op VA**	**HbAIc**				**P value**
	< 6.5%		> 6.5%		
	N	%	N	%	0.032
6/6 - 6/9	16	41	80	31	
6/12 - 6/18	14	36	93	36	
6/24 - 6/36	5	13	42	16	
> 6/60	4	10	46	18	

**Table 4:** Highlights ocular risk factors and treatment patterns across DR stages

**RISK FACTORS**		**N**	**%**
Diabetic retinopathy	No DR	218	72.7
	Mild NPDR	13	4.3
	Moderate NPDR	32	10.7
	Severe NPDR	19	6.3
	PDR	18	6
Ocular association	PXF	28	9.4
	Small Pupil	37	12.3
	Glaucoma	42	14
	ARMD	6	2
	Hard cataract	136	45.3
Treatment	Pan Retinal Photocoagulation	31	10.3
	Intravitreal Anti VEGF	24	8
	Intravitreal Triamcinolone	7	2.3
